# FRET-Based Quantum Dot Immunoassay for Rapid and Sensitive Detection of *Aspergillus amstelodami*

**DOI:** 10.3390/s110606396

**Published:** 2011-06-16

**Authors:** Michele D. Kattke, Elizabeth J. Gao, Kim E. Sapsford, Larry D. Stephenson, Ashok Kumar

**Affiliations:** 1 U.S. Corps of Engineers ERDC-CERL, 2902 Newmark Drive, Champaign, IL 61826, USA; E-Mails: Elizabeth.J.Gao@usace.army.mil (E.J.G.); Larry.D.Stephenson@usace.army.mil (L.D.S.); Ashok.Kumar@usace.army.mil (A.K.); 2 U.S. Food and Drug Administration CDRH-OSEL-DB, 10903 New Hampshire Ave., Silver Spring, MD 20993, USA; E-Mail: kim.sapsford@fda.hhs.gov

**Keywords:** Fluorescence Resonance Energy Transfer (FRET), quantum dot (QD), displacement immunoassay, detection, biosensor, fluorescence, quenching, mold, fungi, spores

## Abstract

In this study, a fluorescence resonance energy transfer (FRET)-based quantum dot (QD) immunoassay for detection and identification of *Aspergillus amstelodami* was developed. Biosensors were formed by conjugating QDs to IgG antibodies and incubating with quencher-labeled analytes; QD energy was transferred to the quencher species through FRET, resulting in diminished fluorescence from the QD donor. During a detection event, quencher-labeled analytes are displaced by higher affinity target analytes, creating a detectable fluorescence signal increase from the QD donor. Conjugation and the resulting antibody:QD ratios were characterized with UV-Vis spectroscopy and QuantiT protein assay. The sensitivity of initial fluorescence experiments was compromised by inherent autofluorescence of mold spores, which produced low signal-to-noise and inconsistent readings. Therefore, excitation wavelength, QD, and quencher were adjusted to provide optimal signal-to-noise over spore background. Affinities of anti-*Aspergillus* antibody for different mold species were estimated with sandwich immunoassays, which identified *A. fumigatus* and *A. amstelodami* for use as quencher-labeled- and target-analytes, respectively. The optimized displacement immunoassay detected *A. amstelodami* concentrations as low as 10^3^ spores/mL in five minutes or less. Additionally, baseline fluorescence was produced in the presence of 10^5^ CFU/mL heat-killed *E. coli* O157:H7, demonstrating high specificity. This sensing modality may be useful for identification and detection of other biological threat agents, pending identification of suitable antibodies. Overall, these FRET-based QD-antibody biosensors represent a significant advancement in detection capabilities, offering sensitive and reliable detection of targets with applications in areas from biological terrorism defense to clinical analysis.

## 1. Introduction

Recent terrorist attacks have reminded us of the immediate risk that biothreat agents pose, as well as the need for fast and accurate detection methods. Fungal species and their mycotoxins are among the growing list of biothreat agents of interest [1]. Molds and fungi are known to cause many adverse effects on human health, whether through development of allergies, most commonly asthma or rhinitis; infection of the body; or toxicity [2]. Immunocompromised individuals, such as those receiving immunosuppressive drugs, uncontrolled diabetics, and advanced AIDS patients, are at an elevated risk for infection, especially deep tissue invasion [3,4]. In addition, patients with invasive fungal infections have increased rates of mortality, as well as extended lengths of hospitalization and increased costs associated with their medical care [5]; a study performed by Menzin *et al*. estimated an additional 735,000 hospital days and $1.89 billion in hospital expenses annually in the United States alone due to invasive fungal infections [5].

Such medical conditions are commonly triggered by the inhalation of mold spores or hyphal fragments, which contain secondary metabolites, called mycotoxins [2]. Mycotoxins play a variety of roles in disease, ranging from carcinogens to immunosuppressants, and are produced mainly by two fungal species: *Aspergillus* and *Penicillium* [6]. *Aspergillus* species produce large numbers of air-borne spores, which infect humans through inhalation and cause chronic mycetoma and invasive aspergillosis, in addition to the previously discussed conditions [6]. One such *Aspergillus* species, called *Aspergillus amstelodami*, has been shown to produce multiple toxins, including Patulin, Ochratoxin A and Sterigmatocystin, as well as elicit symptoms of decreased weight, hair loss, and decreased activity in murine subjects upon exposure [7].

With the health risks of *Aspergillus* species well-defined, the need for positive identification of mold spores in an effort to reduce or eliminate the health risks associated with prolonged exposure still remains. One mode of detection includes monitoring inherent fluorescence of mold spores [8–11]. Intense autofluorescence from fungi following exposure to ultraviolet light has been reported by several research groups [12,13]. Interestingly, each fungus has a characteristic emission spectrum [12], which is dependent upon chemical structure of the endogenous fluorophores present within the cell wall [14]; however, intrinsic autofluorescence of biological samples is not fully understood [14]. Although bioparticle detection via autofluorescence affords real-time analysis [15], such an approach lacks specificity due to numerous non-hazardous autofluorescent biomolecules that can obscure emission profiles of targets of interest.

**Figure 1.**
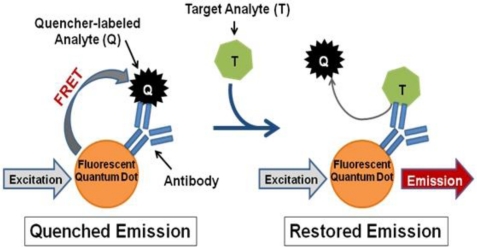
Mechanism of Mold Detection. The initial biosensor complex is formed when a quencher-labeled analyte is bound by the antigen-binding site of the QD-conjugated antibody; when excited, the QD will transfer its energy through FRET to the quencher molecules due to their close proximity. Upon addition of the target analytes, displacement of the quencher-labeled analytes causes disruption of FRET, which translates to increased QD donor emission signal.

In contrast, we propose a fluorescence resonance energy transfer (FRET)-based detection approach involving fluorescent quantum dots (QDs). QDs hold several advantageous properties for labeling applications, such as superior stability against photobleaching in comparison to traditional fluorophores [16,17], compatibility with aqueous environments necessary for biological use [16,18], and capability to concurrently use multiple QDs with different emission wavelengths to produce a multiplexed system [16]. QDs can also be linked to biomolecules, such as antibodies, to produce biosensors capable of fast, sensitive, and specific biological target detection [19]. Such QD-antibody conjugates can be utilized as donors in FRET-based systems with organic quenchers as acceptors for simple and accurate target identification [20–22]. Several FRET-based biosensors have been successfully produced, which operate according to the following mechanism (Figure 1): (1) a quencher-labeled analyte is prebound within the recognition site of the conjugated antibody resulting in FRET quenching of QD fluorescence, then (2) the addition of target analyte displaces the quencher-labeled analyte, which creates an optical signal through restoration of QD fluorescence in a concentration-dependent manner [21,22].

We have developed a system that mimics previous FRET-based QD biosensors in respect to the mechanism of action employed; however, our system is specifically designed for the detection of mold spores in solution. As with all FRET systems, several parameters determine the rate and efficiency of energy transfer, including: (1) quantum yield of the energy donor, (2) spectral overlap of the donor’s emission spectrum with the acceptor’s absorption spectrum, (3) orientation of the donor and acceptor transition dipoles, and (4) the distance between the donor and acceptor molecules [14,23]. In addition to these classical parameters, it was also necessary to address the issue of mold spore autofluorescence to obtain an optimal fluorescence signal. FRET experiments with cell biological samples are frequently hindered by poor signal-to-noise ratios, which forces researchers to analyze results in terms of “FRET” or “no FRET” [23]. This study describes optimization and characterization of a FRET-based displacement immunoassay capable of sensitive and rapid biological target detection and identification.

## 2. Experimental Section

### 2.1. Apparatus and Materials

Solution fluorescence measurements were performed using the Fluoromax 4 spectrofluorometer from Horiba Jobin Yvon (Edison, NJ, USA) with quartz cuvettes from Starna Cells (Atascadero, CA, USA). Fluorescence measurements of sandwich immunoassays were taken with Tecan Infinite M1000 plate reader (Durham, NC, USA). Anti-*Aspergillus* monoclonal detection antibody (IAQ-8602) and capture antibody (IAQ-8601) was obtained from Alexeter Technologies (Rockford, IL, USA). Qdot 625 Antibody Conjugation kits (A10197), Qdot ITK (PEG) quantum dots (Q21541MP, Q21531MP, Q21501MP, A10200, Q21521MP, and Q21561MP), QuantiT protein assays (Q33210), and Lo bind PCR tubes were obtained from Invitrogen Corporation (Carlsbad, CA, USA). All mold spores were produced by and purchased from Assured Bio Labs, LLC (Oak Ridge, TN, USA). Black Hole Quencher (BHQ)-2 (BHQ-2000S-5) and BHQ-3 (BHQ-3000S-5) were obtained from Biosearch Technologies (Novato, CA, USA). Cy5 mono amine reactive—NHS-ester dye (PA25001) was obtained from GE Lifesciences (Piscataway, NJ, USA). Zeba Spin desalting columns (7k MWCO, 2 mL) (89890) were purchased from Pierce-Thermo Scientific (Rockford, IL, USA). Amicon Ultra-4 Ultracel-PL membrane filters (100 kDa, 2 mL) were obtained from Millipore (Billerica, MA, USA). White Corning Costar high binding polystyrene and NBS—low binding 96-well plates were purchased from Fisher Scientific (Pittsburgh, PA, USA). PBS (0.01 M Phosphate Buffer Saline, 138 mM NaCl, 2.7 mM KCl, pH7.4) (P3813), DMSO (D8418), Thermalseal film, Bovine Serum Albumin (BSA) (A7030), and polypropylene slip tip syringes (1 mL, 3 mL) were purchased from Sigma-Aldrich (St. Louis, MO, USA).

### 2.2. QD-Antibody Conjugation

QD-antibody conjugation was performed according to the Qdot Antibody Conjugation Kit procedure from Invitrogen. Briefly, 10 mM succinimidyl-4-(*N*-maleimidomethyl)cyclohexane-1-carboxylate crosslinker (SMCC) was equilibrated to 37 °C for 15 min. Afterwards, 14 μL of thawed SMCC was added to 125 μL of 4 μM amine-derivatized, polyethylene glycol (PEG)-coated CdSe/ZnS Qdot nanocrystals; the solution was vortexed briefly and incubated for 1 h at room temperature in the dark. After 30 min, 300 μL of 1 mg/mL IgG antibody was activated with 20 mM dithiothreitol (DTT), vortexed briefly, and incubated for 30 min at room temperature in the dark. Both solutions were desalted, and 500 μL of each solution was collected in the same tube; during the 1 h reaction, thiols present on the reduced antibodies were coupled to reactive maleimide groups present on the SMCC-activated nanocrystals. The reaction was quenched with 10 μL of 10 mM 2-mercaptoethanol for 30 min. The conjugate was then concentrated to a final volume of 40 μL by centrifuging at 7,000 rpm (4,700 g) for ∼15 min with 50 kDa MWCO filters. The concentrated conjugate was purified using a separation column; ten drops or ∼200 μL of conjugate was collected and stored at 4 °C.

### 2.3. Quantification of Antibody:QD Ratio

The concentration and antibody:QD ratio of the conjugate was analyzed using UV-Vis spectroscopy and QuantiT protein assay from Invitrogen. The conjugate was diluted 1:200 with PBS and analyzed in triplicate for absorbance at 608 nm using a Cary UV-Vis spectrometer. QD concentration, which was assumed to represent conjugate concentration, was calculated using Beer’s law:

$${\text{A}}_{608}\times \text{DF}=ɛ\text{cl}$$

where, ɛ = 500,000 M^−1^cm^−1^ for 625 QDs at the absorbance maximum of 608 nm, c = concentration, DF = dilution factor, and l = path length. Antibody concentration of the conjugate solution was determined according to the QuantiT protein assay protocol; two microliters of conjugate was added to 198 μL of working solution and incubated for 15 min. Triplicate readings from a Qubit fluorometer were averaged to calculate the antibody concentration in units of mg/mL, which was then converted to units of μM assuming antibody molecular weight to be 150,000 g/mol. The average antibody:QD ratio, calculated with the molar concentrations of both components, was compared to the ideal 3–4:1 ratio reported by Invitrogen.

### 2.4. Reduction of Spore Autofluorescence

*Aspergillus* mold species (*A. flavus*, *A. fumigatus*, *P. brevicompactum*, and *P. chyrosegenum*) at 10^6^ spores/mL concentration—with the exception of *A. niger* and *A. amstelodami*, which were 5 × 10^5^ spores/mL and 8.9 × 10^5^ spores/ml, respectively—and solutions of 4 nM QDs were excited at 350–550 nm in 25 nm increments; emission spectra was measured from 400–750 nm. QD emission intensities were divided by the emission intensity of the most autofluorescent mold species at that QD’s wavelength to create a signal-to-noise ratio.

### 2.5. Sandwich Immunoassay

Sandwich immunoassays were performed to analyze spore reactivity with the anti-*Aspergillus* detection antibody. Costar flat-bottom, high binding, white polystyrene 96-well plates were coated with 50 μL of 10 μg/mL anti-*Aspergillus* capture antibody in PBS and incubated overnight at 4 °C. The solution was removed, and wells were washed 4× with 200 μL of water. Two hundred microliters of blocking solution (1% BSA in PBS) was added to each well and incubated for 1 h at room temperature. Blocking solution was removed, and the wells were loaded with 50 μL of diluted mold species, including *A. flavus, A. fumigatus, A. niger, A. amstelodami, Alternaria alternata, Penicillium variable, Rhizopus stolonifer, and Cladosporium cladosporiodes II*, prepared in PBS with 0.1% BSA, which were incubated for 1 h at room temperature. The solution was removed, and wells were washed 4× with 200 μL of PBS. Five hundred microliters of 1 mg/mL Anti-*Aspergillus* detection antibody was reacted with one vial of Cy5 monodye dissolved in 50 μL DMSO for 30 min in the dark. Meanwhile, a 2 mL 7k MWCO Zeba spin column was washed 3× with 1 mL PBS by centrifuging at 1,000 g for 2–3 min; the antibody-Cy5 reaction was loaded into the Zeba spin column and spun at 1,000 g for 2–3 min to collect the labeled antibody. The Cy5-labeled detection antibody was prepared for the assay by diluting the stock to a final concentration of 10 μg/mL using PBS containing 0.1% BSA. Fifty microliters of the prepared anti-*Aspergillus* detection antibody solution was then added to each well and incubated for 1.5 h at room temp. The solution was removed, and wells were washed twice with 200 μL of PBS, followed by two washes with 200 μL of water. Wells were dried with air and measured for Cy5 fluorescence using a fluorescence plate reader.

### 2.6. Quenching Efficiency Experiments

Two hundred microliters of 10^6^ spores/mL *A. fumigatus* was labeled with 25 μg/mL of the quencher species, BHQ-3, for 2 h at room temperature or overnight at 4 °C. BHQ-3-labeled and unlabeled spores were then washed twice by centrifuging at 13,000 rpm (16,200 g) for 10 min, aspirating the supernatant, and resuspending with 50 μL of PBS. One hundred picoliters of QD-antibody conjugate was added to both the BHQ-3-labeled and unlabeled spores and incubated for 30 min. The conjugate-spore complexes were washed as before and suspended to a final volume of 200 μL with PBS. The samples were excited at 450 nm, and emission spectra were measured from 575–675 nm. Intensities at 625 nm of BHQ-3-labeled and unlabeled samples were used to calculate FRET quenching efficiency as follows:

1−(F′D/FD)

where, F’D is the donor emission intensity with the presence of the energy acceptor (BHQ-3-labeled spores) and FD is the donor emission intensity in the absence of the energy acceptor (unlabeled spores).

### 2.7. Displacement Immunoassay

BHQ-3-labeled *A. fumigatus* was prepared as previously described in Section 2.6. Five microliters of QD-antibody conjugate was added for every 10^6^ quenched spores and incubated for 30 min. The *A. fumigatus*-BHQ-3-QD-antibody complex was washed as before and suspended to a final calculated volume to accommodate 250 μL for every 10^6^ spores; 50 μL aliquots were spun down and resuspended immediately before use. Aliquoted volumes of *A. fumigatus*-BHQ-3-QD-antibody complex were added to 150 L of *A. amstelodami* samples at concentrations of 10^2^–10^5^ spores/mL and incubated for 10 min to allow displacement to occur. Samples were excited at 450 nm, and emission spectra were analyzed from 575–675 nm. Time-based displacement immunoassays were performed as described with the exception of the 10 min incubation period; instead, samples were analyzed immediately by reading peak emission intensity every 20 s over a 15 min time period.

## 3. Results and Discussion

### 3.1. QD-Antibody Conjugate Characterization

Purification of QD-antibody conjugates was achieved by gravity flow size-exclusion chromatography according to the Invitrogen protocol. To confirm that antibodies were present in the eluted fraction, and therefore, properly conjugated to QDs, QD-antibody conjugates were analyzed using Invitrogen’s QuantiT protein assay. Additionally, QD composition of the eluted fraction was characterized by UV-Vis spectroscopy, which indicated that QD-antibody conjugate concentration was 1.48 μM on average (Table 1). Concentration of antibodies and QDs in the eluted fraction suggested that QD-antibody conjugates contained ∼4.2 antibodies for every QD; however, this antibody:QD ratio varied across multiple conjugation reactions, perhaps due to slight differences in the volume of activated QDs and reduced antibodies that was collected during each trial. Antibody:QD ratios and conjugate concentration were routinely analyzed for quality control following each conjugation procedure to verify that QD-antibody conjugates were properly formed and purified. Conjugate batches with significantly increased antibody:QD ratios, such as batch 8 (Table 1), indicated the presence of unconjugated antibodies in solution and were discarded; these excess antibodies can compete to bind target during the displacement immunoassay and therefore negatively impact detection sensitivity. Excess, unconjugated antibodies present in such batches may be removed using size-exclusion high performance liquid chromatography (HPLC), although this method was not applied in this study. Conjugate batches whose concentration varies significantly from the average, such as batch 5 (Table 1), were adjusted to maintain consistent sensor complex formation.

### 3.2. FRET Immunoassay Optimization

**Figure 2.**
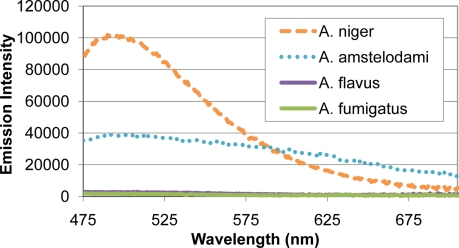
Mold Spore Autofluorescence. Emission spectra and intensity of different *Aspergillus* mold species are shown.

Many mold species have intense autofluorescence, such as *A. niger* and *A. amstelodami*, while others including *A. flavus* and *A. fumigatus* have limited autofluorescence (Figure 2). High autofluorescence can interfere with fluorescence-based detection systems, so it is important to optimize the assay conditions to minimize this potential interference. To determine optimal excitation wavelength for the FRET sensor, QDs (with emission maxima ranging from 525–705 nm) and different mold species (*A. niger*, *A. amstelodami*, *A. flavus*, *A. fumigatus*, *P. brevicompactum*, and *P. chyrosegenum*) were excited individually at various wavelengths in an attempt to minimize mold autofluorescence while maintaining strong QD signal. Peak QD emission intensity was divided by the emission intensity of the most autofluorescent mold species at the peak QD wavelength (*A. niger* or *A. amstelodami*), resulting in signal-to-noise ratios (Table 2). Generally, signal-to-noise ratios for QDs peaked when excited with wavelengths beyond 425 nm.

As expected, less autofluorescence from the mold species was observed as the excitation wavelength increased; according to Hairston *et al.*, shorter excitation wavelengths contain higher energy, and therefore, are more likely to produce fluorescence in different samples (9). The majority of QDs produced optimal signal-to-noise when excited at 450 nm; therefore, a 450 nm excitation wavelength was chosen for all subsequent experiments. The 625 nm QD consistently produced the highest signal-to-noise and was subsequently chosen as the FRET donor.

**Figure 3.**
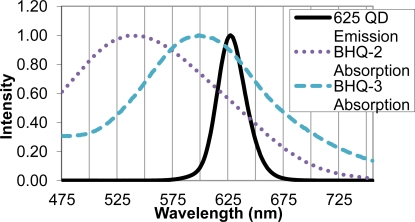
Spectral Overlap of FRET Donor-Acceptor Pair. Emission spectrum of 625 nm QD (FRET donor) and absorption spectra of BHQ-2 and BHQ-3 (possible FRET acceptors) are shown.

Upon identifying the 625 nm QD as an appropriate FRET donor, two quencher molecules, BHQ-2 and BHQ-3, were evaluated as suitable FRET acceptors. Sufficient overlap of the emission spectrum of the FRET donor and absorption spectra of the FRET acceptor is necessary for efficient energy transfer, and in this case, quenching of the QD donor emission. The BHQ-2 absorption spectrum peaked from 525–550 nm, while BHQ-3 showed maximal absorption from 587–613 nm. The absorption spectrum of BHQ-3 offered significantly more overlap with the 625 nm QD emission spectra, as seen in Figure 3, and was therefore chosen as an appropriate quencher for the FRET system.

**Figure 4.**
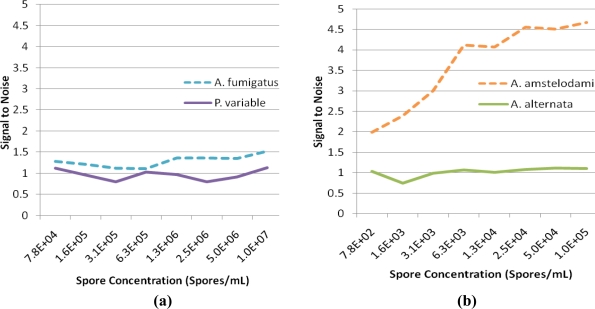
Antibody Reactivity with Different Mold Spores. **(a)** Anti-*Aspergillu*s IgG Affinity for *A. fumigatus. A. fumigatus* was detected with anti-*Aspergillus* antibody in sandwich format. Signal-to-noise values for various concentrations of *A. fumigatus* and *P. variable* are shown for comparison. **(b)** Anti-*Aspergillus* IgG Affinity for *A. amstelodami. A. amstelodami* was detected with anti-*Aspergillus* antibody in sandwich format. Signal-to-noise values for various concentrations of *A. amstelodami* and *A. alternata* are shown for comparison. *A. flavus, A. niger, Rhizopus stolonifer, and Cladosporium cladosporiodes II* produced signals that were indistinguishable from background noise; therefore, they were not included in this figure.

For successful displacement to occur, as outlined in Figure 1, the detection antibody must be prebound to a mold species for which it has lower affinity. When a mold species that has relatively higher affinity for the antibody is present, equilibrium will drive displacement of the lower affinity mode analyte. Sandwich immunoassays were initially performed to characterize the antibody reactivity with different mold species and identify appropriate low affinity (quencher-analyte) and high affinity (target-analyte) analytes for the displacement immunoassay (Figure 4). Of all the mold spores tested, *A. fumigatus* produced a maximum signal-to-noise of 1.53 when detected at 10^7^ spores/mL. In comparison, *A. amstelodami* produced a signal-to-noise of 1.99 with only 8 × 10^2^ spores/mL and achieved values as high as 4.67 with 10^5^ spore/mL concentration. The ability of *A. amstelodami* to produce high signal-to-noise at comparatively low concentrations indicated that the antibody had high affinity for this particular mold species. Therefore, *A. amstelodami* was chosen as a promising target analyte, while *A. fumigatus* was selected for the lower affinity quencher-labeled analyte for the mold-specific antibody.

**Figure 5.**
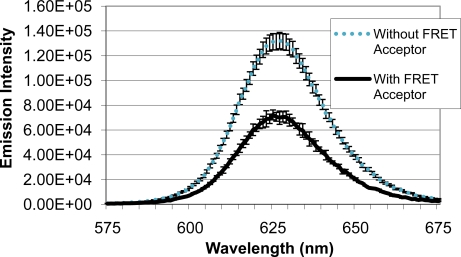
Quenching Efficiency of FRET Detection System. Emission intensity of 625 nm QD-anti-*Aspergillus* conjugate bound to *A. fumigatus* spores labeled (with FRET acceptor) or unlabeled (without FRET acceptor) with BHQ-3 is shown.

Upon identifying a mold species (*A. fumigatus*) and quencher molecule (BHQ-3) for use as the quencher analyte, quenching efficiency of the FRET complex was analyzed (Figure 5). When QD-antibody conjugate was bound to *A. fumigatus* with no BHQ-3, an emission intensity of 131,322 units was produced. Emission intensity of the system decreased to 72,228 units when BHQ-3-labeled *A. fumigatus* was introduced. Average quenching efficiency was calculated to be 0.45, demonstrating the functionality of the FRET complex.

### 3.3. Displacement Immunoassay

**Figure 6.**
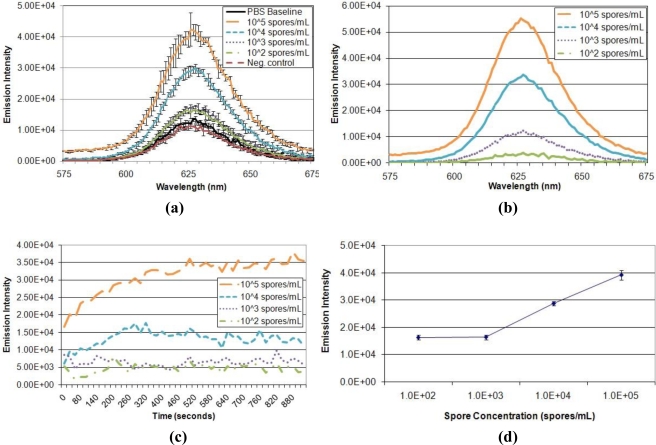
Detection of *A. amstelodami* with FRET-based Displacement Immunoassay. **(a)** Raw Data for Target Detection. Emission Intensity of *A. amstelodami* concentrations and controls are shown. PBS baseline contained sensing complex with PBS instead of a target sample. Heat-killed *E. coli* O157:H7 was used as a negative control. **(b)** Normalized Averaged Data for Target Detection. Emission intensities were first normalized by subtracting the PBS baseline emission intensity and then averaged from multiple trials. **(c)** Development of Emission Signal Over Time. Fluorescence intensity produced at 625 nm by different target concentrations was monitored every 20 s over a period of 15 min. Detection data from multiple trials was averaged and normalized by subtracting the average PBS baseline emission intensity. **(d)** Emission Intensity Dependence on Target Concentration. Average emission intensity at 625 nm is shown with standard deviation error bars.

Having optimized the parameters of the FRET system and verified quenching ability, performance of the displacement immunoassay was evaluated (Figure 6). First, the ability of the assay to detect and distinguish different target concentrations was assessed (Figure 6(a,b)). Exposure to varied *A. amstelodami* concentrations produced increased QD donor emission intensities, all of which were above baseline signal. In addition, exposure to non-targeted *E. coli* O157:H7 maintained baseline fluorescence, demonstrating selectivity of the immunoassay. The fluorescence signal developed very rapidly upon exposure to target analyte, reaching near maximum intensity in less than 5 min for samples of higher target concentration (10^4^ and 10^5^ spores/mL); all target samples, including 10^3^ and 10^2^ spores/mL, produced an immediate increase in signal, as shown by the normalized emission intensity in Figure 6(c). Also, the QD donor emission intensity was found to increase in a dose-dependent manner (Figure 6(d)); as the concentration of *A. amstelodami* increased, more QD donor emission was restored due to displacement of quencher analyte. The lower limit of detection of the FRET-based displacement immunoassay was determined to be 10^3^ spores/mL as indicated by a signal-to-noise of 1.28 (Table 3). This FRET-based displacement immunoassay represents a significant improvement over currently available lateral flow immunoassays, which require a minimum of 15 min to achieve low end limit of detection of 10^5^ spores/mL [24]. Many exposure standards classify indoor spore counts exceeding 10^5^ per cubic meter as a “high level” of mold contamination, although the clinical relevance of this value can be debated due to differences in human susceptibilities and toxicity of different mold species [25].

It is important to note that although 10^3^ spores/mL of *A. amstelodami* was clearly detected and distinguished from other target concentrations, the resulting emission intensity varied among trials. Detection trials with high baseline fluorescence (tens of thousands of intensity units) produced significantly higher intensities in the presence of target compared to trials with lower baseline fluorescence. However, trials with lower baselines (thousands of intensity units) produced more distinguishable signal-to-noise values for different target concentrations when normalized with the baseline. Detection trials that used the same batch of *A. fumigatus*-BHQ-3-QD-antibody complex yielded highly reproducible and consistent QD donor emission signals (see detection trials 6 and 7 in Table 3). In addition, trials using *A. fumigatus*-BHQ-3-QD-antibody complexes that had been prepared separately with the same batch of QD-antibody conjugate (detection trials 1–5) produced fairly similar signal-to-noise, although an instance of enhanced signal-to-noise was noted in trial 5 (Table 3). These observations indicated the importance of normalizing data with the baseline for more accurate analysis, as well as identified aspects of the immunoassay that are in need of refinement.

Signal variation could be due to differences in the antibody:QD ratios of the conjugate batches, as well as variation among quencher-labeled analyte-conjugate complexes. It may be possible that the number of antibodies conjugated to each QD effects the amount of conjugate that is incorporated into the FRET complex during preparation. As more conjugated antibodies are present, quencher-labeled analyte may be bound with less conjugate, resulting in fewer QDs present; these FRET complexes would have lower overall fluorescence compared to complexes formed using conjugate with a low antibody:QD ratio. In contrast, one could argue that all antibody binding sites of the QD-antibody conjugate are not initially bound by quencher-labeled analyte. Conjugate batches with higher antibody:QD ratios contain more unoccupied antibody binding sites that bind target analyte without influencing energy transfer of the FRET system; subsequently, the donor emission signal is not as prominent, producing less signal-to-noise in the presence of target analyte. If methods were developed to ensure consistent QD-antibody conjugation, as well as consistent quencher-labeled analyte-conjugate complex formation, variations in quenching efficiency and QD emission signal could possibly be resolved, facilitating more accurate target quantification and lower detection limits.

## 4. Conclusions

This study documents the development of a FRET-based QD immunoassay for the detection of *A. amstelodami*. The detection complex was formed by conjugating QDs to antibodies and incubating with quencher (BHQ-3)-labeled mold analytes that had a lower affinity for the antibody than *A. amstelodami*. QD-antibody conjugates were first characterized following production and purification in terms of concentration and antibody:QD ratio; these characteristics were used for quality control measures. Complications attributed to mold autofluorescence were minimized by adjusting the excitation wavelength used and strategically selecting a QD that produced large signal-to-noise ratios. Quenching efficiency was optimized by selecting a FRET donor-acceptor pair with maximal spectral overlap. After identifying *A. fumigatus* and *A. amstelodami* for use as quencher and target analytes, respectively, performance of the subsequent FRET complex was analyzed. The optimized displacement immunoassay detected *A. amstelodami* concentrations as low as 10^3^ spores/mL in five minutes or less, while maintaining baseline fluorescence in the presence of 10^5^ CFU/mL heat-killed *E. coli* O157:H7 (negative control). However, it may be possible to improve detection sensitivity and reduce signal variation by identifying a more reliable and consistent method of QD-antibody conjugation and FRET complex formation. If one can control the number of antibodies present on a QD, quenching efficiency can be enhanced, translating to lower detection limits and uniform signal production. Overall, our FRET-based displacement immunoassay offered extremely rapid, sensitive, and selective detection of the fungal species *A. amstelodami*, representing a significant advancement in detection capabilities. In addition, this sensing modality may find great utility in rapid detection and identification of other biological agents of interest; the FRET-based QD-antibody biosensor can be adapted for such targets by incorporating suitable antibodies and surrogates for specific antigens of interest to address detection needs in areas from biological terrorism defense to clinical analysis.

## Figures and Tables

**Table 1.** Composition of QD-antibody Conjugates. The [Ab]:[QD] ratio was calculated by dividing the antibody concentration of conjugate solution by the QD concentration.

Conjugate Batch	Conjugate Concentration (μM)	[Ab]:[QD] Ratio
1	1.3	4.4
2	1.2	3.6
3	1.4	3.1
4	1.1	4.5
5	2.7	1.5
6	1.4	2.6
7	1.7	5.1
8	1.2	7.3
9	1.2	5.8
10	1.6	4.4
Average	**1.48**	**4.20**
Standard Deviation	**0.47**	**1.75**

**Table 2.** Signal-to-Noise Produced with Varied Excitation Wavelengths. Signal-to-noise ratios were calculated for six different QDs of differing fluorescence emissions in the presence of maximum mold spore autofluorescence, at the peak wavelength of each QD of interest. Maximum signal-to-noise ratios for each QD are shown in bold.

Excitation Wavelength (nm)	525 nm	565 nm	605 nm	625 nm	655 nm	705 nm
350	3.8	27.1	125.2	855.6	584.0	**
375	2.4	17.8	83.6	578.4	426.1	196.6
400	1.2	9.0	42.4	323.7	231.7	130.8
425	1.2	9.0	44.1	352.5	224.4	180.7
450	6.0	36.8	177.5	**1,687.2**	**955.1**	**271.4**
475	6.6	69.9	**219.3**	1670.6	898.5	173.3
500	**32.6**	109.6	201.6	804.6	795.9	230.3
525	*	62.3	164.8	731.2	735.5	164.4
550	0.4	**123.6**	182.5	437.4	574.4	84.5

* Emission signal of the 525 nm QD was obscured when excited at 525 nm due to interference from excitation photons.

** When excited at 350 nm, the emission signal of the 705 nm QD was obstructed by an unexplained peak, possibly contributed by QD-solvent interactions that are not well understood.

**Table 3.** Signal-to-Noise of Mold Spore Samples Detected with Displacement Immunoassay. Signal-to-noise values were calculated by dividing the sample fluorescence at 625 nm by the fluorescence produced with PBS only.

**Detection Trial**	10^5^ spores/mL	10^4^ spores/mL	10^3^ spores/mL	10^2^ spores/mL
1	2.07	1.65	1.27	1.01
2	1.73	1.60	1.08	1.01
3	2.20	1.56	1.12	1.03
4	2.10	1.69	1.24	1.15
5	5.21	3.37	1.61	1.31
6	3.23	2.37	1.35	1.34
7	3.19	2.38	1.29	0.97
**Average**	**2.82**	**2.09**	**1.28**	**1.12**
**Standard Deviation**	**1.20**	**0.67**	**0.17**	**0.15**
